# Combination HIV Prevention Strategies in the Lisbon Cohort of Men who Have Sex with Men: A Longitudinal Cluster Analysis of Data from 2014 to 2021

**DOI:** 10.1007/s10461-025-04693-z

**Published:** 2025-04-17

**Authors:** Rita Dias, Miguel Rocha, Luís Veríssimo, Fernando Ferreira, Maria João Novais, Milton Severo, Sílvia Fraga, Paula Meireles

**Affiliations:** 1 EPIUnit ITR, Instituto de Saúde Pública da Universidade do Porto, Universidade do Porto, Rua das Taipas, n° 135, Porto, 4050-600 Portugal; 2 GAT - Grupo de Ativistas em Tratamentos, Lisboa, Portugal

**Keywords:** HIV, Men who have sex with men, HIV prevention

## Abstract

This study aims to examine how HIV prevention tools were used and how they clustered together among men who have sex with men (MSM) testing at a community-based sexual health center in Lisbon, Portugal, from 2014 to 2021. We used data from 16,780 visits from adult cisgender MSM and had an HIV-negative test result at baseline in the Lisbon Cohort of MSM—an open, prospective cohort study conducted at CheckpointLX, a community-based sexual health center tailored to MSM, from 2014 to 2021. A longitudinal clustering approach was used to identify clusters of HIV prevention (*cluster* package for R). Six clusters of HIV prevention were identified: condom use (9,109 visits); low or no condom use, low PrEP (preexposure prophylaxis) use (6,258 visits); anal sex abstinence (746 visits); PEP (postexposure prophylaxis) and condom use (305 visits); PEP use (186 visits); and PrEP and condom use (176 visits). Most participants were aged 24 to 34 years old, were born in Portugal, had high education, and self-identified as gay. PrEP and PEP uptake were more associated with being born in Brazil, while low prevention adherence was less associated with high education. Condom use was the most frequently reported prevention strategy, followed by the low or no condom use, low PrEP use cluster. However, participants with higher odds of reporting HIV risk behaviors were the ones allocated to clusters with reports of higher frequency of prevention tools utilization.

## Background

In 2022, an estimated 39 million people were living with HIV worldwide, according to the most recent data from the Joint United Nations Programme on HIV/AIDS (UNAIDS). HIV prevalence among men who have sex with men (MSM) was estimated to be significantly higher than in the general adult population aged 15 to 49 years, with UNAIDS reporting that MSM had 11 times higher HIV prevalence than the general adult population [1]. In Portugal, 804 new HIV diagnoses were recorded in 2022, with 91.9% attributed to sexual transmission. Among men, 61.8% of cases were attributed to sex between men [2]. Gay, bisexual, and other MSM continue to experience a disproportionate burden of HIV, with prevalence rates significantly higher than those in the general population, as reported by UNAIDS [1] due to a set of social, political, structural, and biological determinants that make them more susceptible to the infection and, therefore, an important population to the HIV response [3]. In this context, UNAIDS has proposed an integrated and combined strategy to end the HIV epidemic by 2030. This strategy encompasses biomedical and behavioral interventions at individual, social, political, and structural levels, putting key populations at the center [3–5].

Several tools have been proven effective in preventing sexual transmission of HIV. These evidence-based strategies include consistent condom use, preexposure prophylaxis (PrEP), postexposure prophylaxis (PEP), treatment as prevention through antiretroviral treatment, and the prevention, diagnosis, and treatment of other sexually transmitted infections (STIs), along with regular HIV testing and counseling enabling early diagnosis and treatment [6–10]. Gay, bisexual, and other MSM also use other prevention tools that are not recommended to use alone, such as voluntary medical male circumcision, or whose effectiveness has no evidence, such as serosorting, seropositioning, viral-load sorting, or withdrawal before ejaculation. Nevertheless, when these blocks of biomedical and behavioral prevention tools are used correctly and combined, the prevention effects are optimized [11–16].

At an individual level, combination HIV prevention has been conceptualized as combining 2 or more prevention tools or adopting different strategies according to a specific time or life context [14, 17–19]. Most studies about HIV prevention strategies used among gay, bisexual, and other MSM tend to focus only on a single strategy, on a combination of 2 methods, or risk compensation [20]. Other studies have used statistical approaches to describe patterns of HIV prevention utilization, all of them highlighting condom use on one side and low prevention adherence on the other [20–22]. In Portugal, the uptake of HIV prevention strategies among gay, bisexual, and other MSM is relatively well known. Data from 2011 to 2014 from the Lisbon Cohort of MSM showed that most participants had at least 1 HIV-negative steady partner (67.0%) and used condoms more often in the last sexual encounter with an occasional partner (78.3%) than with a steady partner (55.1%) [23]. Between March 2014 and July 2019, 3.2% of the cohort participants reported recent PrEP uptake, increasing its utilization from 0.15% in 2014 to 5.36% in 2019 [23]. Results from the European Men Who Have Sex With Men Internet Survey demonstrated that in Lisbon, 50–59% of MSM reported having knowledge about PrEP [24]. However, PrEP uptake remained low, with 1.5% reporting utilization. Moreover, 23.9% reported 2 condomless anal sex encounters with an occasional partner with unknown HIV status, 14.2% reported condomless anal intercourse with more than 2 steady partners in the previous 12 months, and 14.1% reported not having enough agency towards safer sex practices [24]. In 2019, 1,252 people received PrEP in Portugal at least once, and of those, 52% were MSM [25].

How HIV prevention tools are used and combined has not yet been explored in Portugal. Therefore, we aimed to identify clusters of HIV prevention using anal sex abstinence, consistent condom use, utilization of PrEP and PEP among HIV-negative MSM who were tested at CheckpointLX from 2014 to 2021. We also measured the association between sociodemographic characteristics, behavioral factors, and sexual practices related to HIV risk and the identified clusters of HIV prevention.

## Methods

### Study Design and Participant Selection

The Lisbon Cohort of MSM is an open, noninterval, prospective cohort study conducted at GAT CheckpointLX, a community-based sexual health center tailored to MSM. A detailed description was provided elsewhere [23]. The cohort was established in April 2011 and is ongoing. MSM attending GAT CheckpointLX for HIV testing and who meet the cohort eligibility criteria—being a cisgender man; reporting having or having had sex with men; being 18 years or older; and having an HIV-negative test result at baseline—are invited to participate in the cohort study. The follow-up visits ideally occur every 6 months. However, they occur primarily depending on participants’ visits to GAT CheckpointLX. At each visit, a face-to-face interview using a structured questionnaire is conducted, and trained peer community health workers perform HIV, hepatitis B virus (HBV), hepatitis C virus (HCV), and syphilis point-of-care rapid tests [23]. Linkage to treatment is offered in case of any reactive test result, and when testing reactive for HIV, participants are no longer followed up in the cohort study, regardless of the use of GAT CheckpointLX services [23].

From March 2014 to March 2021, cohort participants made 21,707 eligible visits. However, after excluding visits with missing information for two or more prevention strategies, 16,792 visits were considered for analysis.

The Lisbon Cohort of MSM obtained ethical approval from the Ethics Committee for Health of São João Hospital Center and Medical School, University of Porto. The study follows the Declaration of Helsinki Ethical Principles for Medical Research in Humans. Participants provide informed consent at each visit, and those with reactive test results are offered referral and linkage to treatment and care [23].

### Data Collection and Variables Definition

This study considered 4 main HIV prevention strategies: anal sex abstinence, condom use, PrEP use, and PEP use. Anal sex abstinence was defined as reporting not having had anal sex regardless of the type of partner or the involvement in other sexual practices. Condom use was defined based on the frequency of condom utilization in anal sex with steady and occasional partners. It was categorized as always, almost always, occasionally/rarely/never, and did not have anal sex. It was considered that PEP and PrEP were used when participants reported their utilization. To define these variables, we used the information collected in baseline and follow-up questionnaires corresponding to the last 12 months or the time since the previous visit.

Regarding covariates, the following sociodemographic information was included: age strata divided into 18–24, 25–34, 35–44 stratums; educational level divided into 2 categories (less than higher education and higher education); and the country of birth categorized as Portugal, Brazil, and other. Other behavioral and health-related information included knowledge about PEP measured at baseline; knowledge about PrEP measured at baseline; the number of occasional partners (0; 1–5, and > 5); having had at least 1 steady partner living with HIV (yes, no/does not know); having had at least 1 occasional partner living with HIV (yes, no/does not know); STI diagnosis; sex work defined as sexual intercourse in exchange for money, goods, or drugs; and chemsex, here defined as using gamma-hydroxybutyrate/gamma-butyrolactone (GHB/GBL), methamphetamines, or mephedrone related to sexual practices before or during sex [26]. Behavioral and health-related information, except knowledge about PEP and PrEP, was reported for the last 12 months or the time since the previous visit, depending on the type of visit.

### Statistical Analysis

To aggregate participants in similar patterns of use of preventive strategies we used a cluster analysis. We initially considered both a probabilistic cluster analysis (latent class analysis) assuming that participants could be in more than one pattern and a hierarchical cluster analysis assuming that participants could only be in one pattern. However, for the probabilistic cluster analysis we would only be able to accommodate a maximum of four patterns considering that only 32 combinations were possible (three binary items– Anal sex abstinence, PEP and PrEP use– and one with four classes– condom use). On the other hand, the hierarchical cluster analysis allowed us to have more than four patterns which we found to be of more meaningful interpretation; therefore, we opted for this solution. The hierarchical clusters were constructed using the Gower distance to compute all the pairwise dissimilarities given that variables were either categorical or ordered. The complete agglomeration method was used to perform the hierarchical cluster analysis. The dendrogram heights were charted, representing the hierarchical clustering process with the height of the branches indicating the level of dissimilarity between data points or clusters (fusion coefficients). The fusion coefficients were used to determine the optimal number of clusters by identifying significant jumps in the dendrogram heights.

For the hierarchical cluster analysis, and considering that participants could have more than one visit (the median number was 1; IQR: 1–3), data were reshaped to a long format to allow us a longitudinal analysis, each row representing a unique observation (each single visit in the cohort from included participants). We first tested the configural time invariance of clusters by comparing the number and interpretability of the identified clusters using all visits, using only the first visit and using only the second visit. As the number and interpretability of the clusters were similar, we opted to use data from all visits assuming two participants in the same non-observed cluster would have the same observed response to the preventive strategies independently of the visit [27].

Descriptive statistics were used to characterize participants in each cluster using absolute and relative frequencies.

A multinomial logistic regression was performed using generalized linear mixed models via penalized quasi-likelihood with a random intercept per participant to assess the associations between the covariates mentioned above and the clusters of HIV prevention identified, assuming missing at random. For the time-invariant covariates the value reported at the first visit was used for all subsequent observations. Adjusted odds ratios (aORs) and 95% confidence intervals (95% CIs) were computed. The associations of age, country of birth, and educational level were adjusted for each other, and the remaining covariates were adjusted for age, country of birth, and educational level. The *cluster* R version 2.0 7 − 1 and *mass* for R packages were used for statistical analysis (R version 4) [28, 29].

## Results

**Fig. 1:**
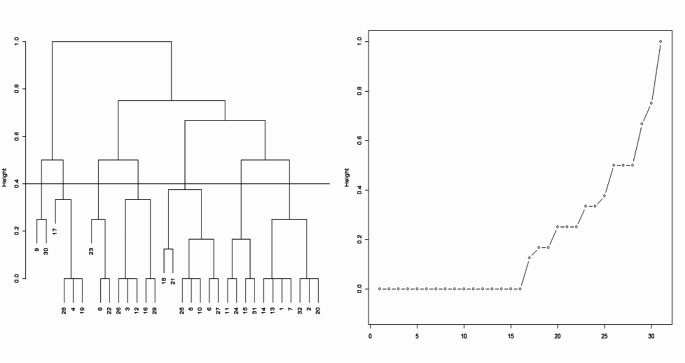
shows the aggregated response patterns from Hierarchical clustering analysis with Gower Distance using the complete agglomeration method. The horizontal line shows the cut-off value for deciding the number of clusters of possible HIV Prevention Strategies: Cluster Dendrogram (**a**) and Fusion Plot (**b**)

The analysis of the cluster dendrogram and fusion plot (Fig. 1) suggested the identification of 7 HIV prevention clusters with 16,792 observations. However, we excluded 1 cluster with less than 0.1% of the total observations (*n* = 12). Therefore, 16,780 observations from 7,549 participants were considered for analysis.

### Prevention Strategies Distribution Per Cluster

Six different clusters of HIV prevention strategies were identified: *condom use* (cluster 1), *low or no condom use*,* low PrEP use* (cluster 2), *anal sex abstinence* (cluster 3), *PEP and condom use* (cluster 4), *PEP use* (cluster 5), and *PrEP and condom use* (cluster 6). The distribution of prevention strategies per cluster is presented in Table 1. The condom use cluster was the most frequent, with 9,109 visits (54.3%). It was characterized by having used condoms always (50.7%) or almost always (46.8%) and not reporting other strategies. In the low or no condom use, low PrEP use cluster, 6,258 visits (33.3%) were made. This cluster was characterized by participants reporting using condoms occasionally, rarely, or never (100%), and, although much less frequent, by reporting to have used PrEP (4.5%; 281 visits). In the cluster of anal sex abstinence, participants reported to have abstained from anal sex (100%; 746 visits). In the cluster of PEP and condom use, there were 305 visits. In this cluster, participants reported PEP use (100%) and having used condoms almost always (53.4%; 163 visits) or always (43.6%; 133 visits). In the PEP use cluster, participants reported only PEP use (100%; 186 visits). In the cluster of PrEP and condom use, participants reported having used PrEP (100%; 176 visits) and having used condoms almost always (64.2%; 113 visits) or always (30.7%; 54 visits) (Table 1).

**Table 1:** HIV prevention strategy allocation by cluster

	Clusters *n* (%)							Χ^2^Statistic	df	*p*-value
	Overall	Cluster 1	Cluster 2	Cluster 3	Cluster 4	Cluster 5	Cluster 6			
Number of visits		*n* = 9,109	*n* = 6,258	*n* = 746	*n* = 305	*n* = 186	*n* = 176			
**HIV PREVENTION STRATEGIES**										
**Anal sex abstinence in the last 12 months**								105.28	15	< 0.001
Yes	746 (4.4)	0	0	**746 (100.0)**	0	0	0			
No	16,034 (95.6)	9,109 (100.0)	6,258 (100.0)	0	305 (100.0)	186 (100.0)	176 (100.0)			
**Condom use in anal sex in the last 12 months**								99.52	10	< 0.001
Always	4,809 (28.7)	**4**,**622 (50.7)**	0	0	**133 (43.6)**	0	**54 (30.7)**			
Almost always	4,541 (27.1)	**4**,**265 (46.8)**	0	0	**163 (53.4)**	0	**113 (64.2)**			
Occasionally/rarely/never	6,444 (38.4)	0	**6**,**258 (100.0)**	0	0	**186 (100.0)**	0			
Did not have anal sex	746 (4.4)	0	0	**746 (100.0)**	0	0	0			
Missing	240 (1.4)	222 (2.4)	0	0	9 (3.0)	0	9 (5.1)			
**PrEP use in the last 12 months**								99.52	10	< 0.001
Yes	495 (2.9)	0	**281 (4.5)**	6 (0.8)	**17 (5.6)**	**15 (8.1)**	**176 (100.0)**			
No	16,207 (96.6)	9,074 (99.6)	5,946 (95.0)	735 (98.5)	284 (93.1)	168 (90.3)	0			
Missing	78 (0.5)	35 (0.4)	31 (0.5)	5 (0.7)	4 (1.3)	3 (1.6)	0			
**PEP use in the last 12 months**								99.52	10	< 0.001
Yes	491 (2.9)	0	0	0	**305 (100.0)**	**186 (100.0)**	0			
No	16,257 (96.9)	9,102 (99.9)	6236 (99.6)	744 (99.7)	0	0	175 (99.4)			
Missing	32 (0.2)	7 (0.1)	22 (0.4)	2 (0.3)	0	0	1 (0.6)			

*HIV* human immunodeficiency virus, *PEP* postexposure prophylaxis, *PrEP* preexposure prophylaxis

### Visits Allocation Per Cluster Per Year

Table 2 shows the distribution of observations across clusters for the years considered in this analysis. Despite condom use having the highest number of observations overall, its relative importance has declined. In 2014, it accounted for 61.6% of observations, decreasing to 38.7% by 2021. Conversely, low or no condom use, low PrEP use had an increase in relative importance, rising from 29.8% in 2014 to 49.8% in 2021. The clusters of anal sex abstinence, PEP and condom use, and PEP use were reported less frequently and generally remained stable or decreased over time. It is noteworthy that the PrEP and condom use cluster, while having a smaller number of observations in 2014 (0.1%), showed a notable increase in relative importance by 2021 (5.0%).

**Table 2:** Visits per year allocated to the respective cluster

Year	Total number of visits	Cluster					
		Cluster 1 (condom use)	Cluster 2 (low or no condom use, low PrEP use)	Cluster 3 (anal sex abstinence)	Cluster 4 (PEP and condom use)	Cluster 5 (PEP use)	Cluster 6 (PrEP and condom use)
		*n* (%)					
2014	**1**,**663**	1025 (61.6)	496 (29.8)	115 (6.9)	19 (1.1)	6 (0.4)	2 (0.1)
2015	**2**,**288**	1328 (58.0)	761 (33.3)	128 (5.6)	47 (2.1)	13 (0.6)	11 (0.5)
2016	**2**,**687**	1519 (56.5)	929 (34.6)	139 (5.2)	48 (1.8)	30 (1.1)	22 (0.8)
2017	**2**,**755**	1562 (56.7)	978 (35.5)	89 (3.2)	68 (2.5)	41 (1.5)	17 (0.6)
2018	**2**,**597**	1396 (53.8)	1006 (38.7)	107 (4.1)	44 (1.7)	23 (0.9)	21 (0.8)
2019	**2**,**695**	1341 (49.8)	1116 (41.4)	106 (3.9)	45 (1.7)	43 (1.6)	44 (1.6)
2020	**1**,**679**	777 (46.3)	765 (45.6)	48 (2.9)	27 (1.6)	24 (1.4)	38 (2.3)
2021	**416**	161 (38.7)	207 (49.8)	14 (3.4)	7 (1.7)	6 (1.4)	21 (5.0)

### Participants’ Characteristics at Each Visit by Cluster Allocation

Table 3 shows participants’ characteristics at each visit overall and by cluster allocation. Across all clusters, participants were mainly aged between 24 and 34 years, born in Portugal, had high educational levels, and self-identified as gay. Knowledge about PEP varied from 47.3% in the anal sex abstinence cluster to 91.7% in the PEP use cluster, while knowledge about PrEP varied from 31.0% in the anal sex abstinence cluster to 77.3% in the cluster of PEP use. Condom use and, low or no condom use, low PrEP use were reported more often in visits where participants reported having between 1 and 5 occasional partners. In contrast, in the clusters of PEP and condom use, PEP use, and PrEP and condom use, participants reported more than 5 occasional partners.

**Table 3:** Participants’ sociodemographic and behavioral characterization by cluster

Participants’ characteristics at each visit	Overall	Clusters					
		Cluster 1 (condom use)	Cluster 2 (low or no condom use, low PrEP use)	Cluster 3 (anal sex abstinence)	Cluster 4 (PEP and condom use)	Cluster 5 (PEP use)	Cluster 6 (PrEP and condom use)
	No. of visits = 16,780	No. of visits = 9,109	No. of visits = 6,258	No. of visits = 746	No. of visits = 305	No. of visits = 186	No. of visits = 176
**Age strata**,** y**							
18–24	4,117 (24.7)	2,329 (25.6)	1,449 (23.2)	205 (27.5)	75 (24.6)	42 (22.6)	17 (9.7)
25–34	7,061 (42.4)	3,936 (43.2)	2,665 (42.6)	265 (35.5)	152 (49.8)	90 (48.4)	73 (41.5)
35–44	3,534 (21.2)	1,843 (20.2)	1,395 (22.3)	142 (19.0)	60 (19.7)	38 (20.4)	56 (31.8)
≥ 45	1,948 (11.7)	1,001 (11.0)	749 (12.0)	134 (18.0)	18 (5.9)	16 (8.6)	30 (17.0)
*Missing*	*120*	*0*	*0*	*0*	*0*	*0*	*0*
**Country of birth**							
Portugal	11,367 (73.3)	6,404 (74.5)	4,285 (72.8)	595 (8.3)	180 (65.9)	102 (60.7)	101 (60.5)
Brazil	1,970 (12.7)	1,025 (12.0)	789 (13.4)	42 (0.6)	51 (18.7)	40 (23.8)	23 (13.8)
Other^a^	2,173 (14.0)	1,173 (13.6)	810 (13.8)	79 (1.1)	42 (15.4)	26 (15.5)	43 (25.8)
*Missing*	*1270*	*507*	*374*	*30*	*32*	*18*	*9*
**Educational level**							
Less than higher education	6,225 (37.4)	3,333 (37.0)	2,410 (38.8)	269 (36.5)	108 (36.0)	67 (36.2)	38 (22.9)
Higher education	10,402 (62.6)	5,694 (63.1)	3,796 (61.2)	468 (63.5)	192 (64.0)	118 (63.8)	134 (78.0)
*Missing*	*153*	*82*	*52*	*9*	*5*	*1*	*4*
**Sexual Orientation**							
Not gay	1,860 (15.6)	1,039 (16.7)	642 (14.4)	114 (25.7)	34 (16.3)	11 (8.3)	20 (17.0)
Gay	10,051 (84.4)	5,196 (83.3)	3,831 (85.7)	330 (74.3)	175 (83.7)	121 (91.7)	98 (83.5)
*Missing*	*4869*	*2874*	*1785*	*302*	*96*	*54*	*58*
**Knowledge about PEP at baseline**							
No	5,857 (50.0)	3,133 (50.4)	2,289 (51.4)	235 (52.7)	55 (26.4)	30 (22.7)	45 (38.5)
Yes	5,850 (50.0)	3,087 (49.6)	2,161 (48.6)	211 (47.3)	153 (73.6)	102 (77.3)	72 (61.5)
*Missing*	*5073*	*2889*	*1808*	*300*	*97*	*54*	*59*
**Knowledge about PrEP at baseline**							
No	6,867 (59.8)	3,754 (60.5)	2,666 (60.0)	307 (69.0)	108 (52.4)	57 (43.2)	45 (38.1)
Yes	4,611 (40.2)	2,450 (39.5)	1,777 (40.0)	138 (31.0)	98 (47.6)	75 (56.8)	73 (61.9)
*Missing*	*5302*	*2905*	*1815*	*301*	*99*	*54*	*58*
**Number of occasional anal sex partners in the last 12 months**							
0	2,918 (18.4)	1,290 (14.3)	1,632 (26.4)	726 (100)	32 (10.8)	23 (13.3)	15 (8.7)
1–5	7,049 (44.5)	4,264 (47.2)	2,608 (42.2)	0	112 (37.7)	57 (33.0)	58 (33.7)
More than 5	5,856 (37.0)	3,487 (38.6)	1,944 (31.4)	0	153 (51.2)	93 (53.8)	99 (57.6)
*Missing*	*957*	*68*	*74*	*20*	*8*	*13*	*4*
**Steady partner in the last 12 months living with HIV**							
No/does not know	14,410 (92.3)	8,269 (93.8)	5,450 (90.7)	711 (96.3)	258 (86.6)	160 (89.9)	162 (94.2)
Yes	1,201 (7.7)	545 (6.2)	561 (9.3)	27 (3.7)	40 (13.4)	18 (10.1)	10 (5.8)
*Missing*	*1169*	*295*	*247*	*8*	*7*	*8*	*4*
**Occasional partner in the last 12 months living with HIV**							
No/does not know	15,758 (94.1)	8,542 (94.0)	5,917 (94.8)	746 (100)	257 (84.3)	145 (79.2)	151 (85.8)
Yes	982 (5.9)	549 (6.0)	322 (5.2)	0	48 (15.7)	38 (20.8)	25 (14.2)
*Missing*	*40*	*18*	*19*	*0*	*0*	*3*	*0*
**Sex work in the last 12 months**							
No	16,435 (98.1)	8,938 (98.3)	6,117 (97.9)	737 (98.9)	293 (96.4)	180 (96.8)	170 (96.6)
Yes	313 (1.9)	153 (1.7)	129 (2.1)	8 (1.1)	11 (3.6)	6 (3.2)	6 (3.4)
*Missing*	*32*	*18*	*12*	*1*	*1*	*0*	*0*
**Use of GHB/GBL**,** methamphetamines**,** or mephedrone associated with sex (chemsex) in the last 12 months**							
No	16,081 (96.4)	8,861 (97.7)	5,884 (94.8)	737 (99.6)	288 (95.1)	151 (83.0)	160 (91.4)
Yes	600 (3.6)	211 (2.3)	325 (5.2)	3 (0.4)	15 (5.0)	31 (17.0)	15 (8.6)
*Missing*	*99*	*37*	*49*	*6*	*2*	*4*	*1*
**STI diagnosis in the last 12 months**							
No	14,752 (88.5)	8,141 (90.0)	5,388 (86.8)	694 (93.4)	251 (82.8)	145 (78.4)	133 (76.4)
Yes	1,919 (11.5)	914 (10.0)	823 (13.3)	49 (6.6)	52 (17.2)	40 (21.6)	41 (23.6)
*Missing*	*109*	*54*	*47*	*3*	*2*	*11*	*2*

*GHB/GBL* gamma-hydroxybutyrate/gamma-butyrolactone, *HIV* human immunodeficiency virus, *PEP* postexposure prophylaxis, *PrEP* preexposure prophylaxis, *STI* sexually transmitted infection

^a^Other countries include Andorra, Angola, Argentina, Australia, Austria, Bangladesh, Belarus, Belgium, Bolivia (Plurinational State of), Bosnia and Herzegovina, Botswana, Brazil, Bulgaria, Cabo Verde, Canada, Chile, China, Colombia, Costa Rica, Croatia, Cuba, Curacao, Cyprus, Czech Republic, Democratic Republic of the Congo, Denmark, Ecuador, Egypt, El Salvador, Estonia, Faroe Islands, Finland, France, Germany, Greece, Guatemala, Guinea, Guinea-Bissau, Honduras, Hungary, India, Indonesia, Iran (Islamic Republic of), Ireland, Israel, Italy, Jamaica, Japan, Kenya, Kuwait, Kyrgyzstan, Lebanon, Luxembourg, Macau, Malaysia, Mauritius, Mexico, Moldova (Republic of), Morocco, Mozambique, Namibia, Nepal, the Netherlands, Nicaragua, North Korea (Democratic People’s Republic of Korea), Norway, Oman, Pakistan, Palestine (State of), Papua New Guinea, Peru, Philippines, Poland, Portugal, Qatar, Romania, Russian Federation, Sao Tome and Principe, Senegal, Serbia, Singapore, Slovakia, Slovenia, South Africa, Spain, Suriname, Sweden, Switzerland, Syrian Arab Republic, Tanzania, Timor-Leste, Trinidad and Tobago, Tunisia, Turkey, Ukraine, United Arab Emirates, United Kingdom of Great Britain and Northern Ireland, United States of America, Uruguay, Uzbekistan, Venezuela (Bolivarian Republic of), Zambia, and Zimbabwe

### Associations between Participants’ Sociodemographic and Behavioral Factors and Cluster Allocation

Table 4 presents the associations between sociodemographic and behavioral characterization and cluster allocation. The odds of being allocated to the low or no condom use, low PrEP use cluster were higher for individuals in older age groups, born in Brazil (aOR = 1.17; 95% CI: 1.01–1.34) compared with those born in Portugal, who self-identified as gay (aOR = 1.22; 95% CI: 1.05–1.41), who had a steady partner living with HIV (aOR = 1.52; 95% CI: 1.30–1.78), who reported chemsex (aOR = 2.36; 95% CI: 1.90–2.92), and who had an STI diagnosis (aOR = 1.32; 95% CI: 1.17–1.48). On the other side, the odds were lower for those with higher education (aOR = 0.79; 95% CI: 0.71–0.88), who had between 1 and 5 occasional partners (aOR = 0.44; 95% CI: 0.40–0.49) or more than 5 occasional partners (aOR = 0.35; 95% CI: 0.31–0.40), and who had an occasional sex partner living with HIV (aOR = 0.77; 95% CI: 0.65–0.91).

**Table 4:** Associations between clusters of HIV prevention and sociodemographic and behavioral covariates, adjusted odds ratios with 95% confidence intervals

Participants’ characteristics at each visit	aOR (95% CI)					
	Cluster 1 (condom use)	Cluster 2 (low or no condom use, low PrEP use)	Cluster 3 (anal sex abstinence)	Cluster 4 (PEP and condom use)	Cluster 5 (PEP use)	Cluster 6 (PrEP and condom use)
**Age strata, y** ^a^						
18–24	Ref.	Ref.	Ref.	Ref.	Ref.	Ref.
25–34	Ref.	1.27 (1.13–1.43)**	0.80 (0.65–0.99)*	0.99 (0.77–1.27)	1.51 (1.17–1.96)*	1.91 (1.66–2.20)**
35–44	Ref.	1.44 (1.25–1.66)**	0.64 (0.48–0.86)*	0.67 (0.47–0.94)*	1.38 (0.94–2.02)	2.78 (2.37–3.27)**
≥ 45	Ref.	1.42 (1.19–1.70)**	1.07 (0.75–1.52)	0.55 (0.34–0.92)*	0.54 (0.30–0.97)*	3.11 (2.56–3.77)**
**Country of birth (baseline)**						
Portugal	Ref.	Ref.	Ref.	Ref.	Ref.	Ref.
Brazil	Ref.	1.17 (1.01–1.34)*	0.42 (0.29–0.62)**	1.90 (1.27–2.86)*	2.69 (1.79–4.05)**	1.36 (1.16–1.59)**
Other^b^	Ref.	0.96 (0.84–1.11)	0.63 (0.45–0.87)*	1.52 (1.00–2.29)*	1.44 (0.92–2.25)	1.63 (1.42–1.88)**
**Educational level (baseline)**						
Less than higher education	Ref.	Ref.	Ref.	Ref.	Ref.	Ref.
Higher education		0.79 (0.71–0.88)**	0.96 (0.76–1.23)	1.30 (0.93–1.80)	1.04 (0.73–1.46)	1.34 (1.19–1.52)**
**Sexual orientation (baseline)**						
Not gay	Ref.	Ref.	Ref.	Ref.	Ref.	Ref.
Gay	Ref.	1.22 (1.05–1.41)*	0.59 (0.42–0.82)*	1.22 (0.69–2.16)	2.48 (1.18–5.21)*	1.11 (0.63–1.96)
**Knowledge about PEP at baseline**						
No	Ref.	Ref.	Ref.	Ref.	Ref.	Ref.
Yes	Ref.	0.94 (0.84–1.04)*	0.94 (0.72–1.24)*	2.54 (1.33–4.82)*	3.29 (1.11–9.77)*	1.76 (1.16–2.69)*
**Knowledge about PrEP at baselinec**						
No	Ref.	Ref.	Ref.	Ref.	Ref.	Ref.
Yes	Ref.	1.09 (0.97–1.22)	0.72 (0.54–0.95)*	0.98 (0.66–1.45)	2.12 (1.71–2.62)**	2.90 (2.03–4.12)**
**Number of occasional anal sex partners in the last 12 months**						
0	Ref.	Ref.	Ref.	Ref.	Ref.	Ref.
1–5	Ref.	0.44 (0.40–0.49)**	NA	1.01 (0.87–1.18)	0.60 (0.51–0.71)**	0.95 (0.78–1.15)
More than 5	Ref.	0.35 (0.31–0.40)**	NA	1.58 (1.35–1.85)**	1.02 (0.86–1.20)	1.57 (1.29–1.92)**
**Steady partner in the last 12 months living with HIV**						
No/does not know	Ref.	Ref.	Ref.	Ref.	Ref.	Ref.
Yes	Ref.	1.52 (1.30–1.78)**	0.69 (0.50–0.96)*	2.05 (1.70–2.48)**	1.81 (1.41–2.30)**	0.83 (0.69–1.00)*
**Occasional partner in the last 12 months living with HIV**						
No/does not know	Ref.	Ref.	NA	Ref.	Ref.	Ref.
Yes	Ref.	0.77 (0.65–0.91)*	NA	3.22 (2.71–3.84)**	1.72 (1.44–2.06)**	2.26 (1.97–2.61)**
**Sex work in the last 12 months**						
No	Ref.	Ref.	Ref.	Ref.	Ref.	Ref.
Yes	Ref.	1.18 (0.88–1.58)	0.36 (0.18–0.74)*	1.56 (1.00–2.43)*	0.30 (0.17–0.51)**	2.46 (1.82–3.31)**
**Use of GHB/GBL**,** methamphetamines**,** or mephedrone associated with sex (chemsex) in the last 12 months**						
No	Ref.	Ref.	Ref.	Ref.	Ref.	Ref.
Yes	Ref.	2.36 (1.90–2.92)**	0.23 (0.09–0.58)*	6.23 (4.17–9.31)**	9.50 (7.25–12.44)**	3.48 (2.81–4.32)**
**STI diagnosis in the last 12 months**						
No	Ref.	Ref.	Ref.	Ref.	Ref.	Ref.
Yes	Ref.	1.32 (1.17–1.48)**	0.83 (0.69–1.00)	2.10 (1.76–2.49)**	2.09 (1.76–2.48)**	2.79 (2.42–3.21)**

*aOR* adjusted odds ratio, *CI* confidence interval, *GHB/GBL* gamma-hydroxybutyrate/gamma-butyrolactone, *HIV* human immunodeficiency virus, *NA* Not Applicable, *PEP* postexposure prophylaxis, *PrEP* preexposure prophylaxis, *STI* sexually transmitted infection. p values using a notation to report a conventional equivalent, * for *p* <.05 and ** for *p* <.001

^a^Because the n for category 4 in the age class was too small for the knowledge about PrEP at the baseline in cluster 5, for this variable, it was considered as having only 3 age classes, with 35–44 and 45 plus in the same category

^b^Other countries include Andorra, Angola, Argentina, Australia, Austria, Bangladesh, Belarus, Belgium, Bolivia (Plurinational State of), Bosnia and Herzegovina, Botswana, Brazil, Bulgaria, Cabo Verde, Canada, Chile, China, Colombia, Costa Rica, Croatia, Cuba, Curacao, Cyprus, Czech Republic, Democratic Republic of the Congo, Denmark, Ecuador, Egypt, El Salvador, Estonia, Faroe Islands, Finland, France, Germany, Greece, Guatemala, Guinea, Guinea-Bissau, Honduras, Hungary, India, Indonesia, Iran (Islamic Republic of), Ireland, Israel, Italy, Jamaica, Japan, Kenya, Kuwait, Kyrgyzstan, Lebanon, Luxembourg, Macau, Malaysia, Mauritius, Mexico, Moldova (Republic of), Morocco, Mozambique, Namibia, Nepal, the Netherlands, Nicaragua, North Korea (Democratic People’s Republic of Korea), Norway, Oman, Pakistan, Palestine (State of), Papua New Guinea, Peru, Philippines, Poland, Portugal, Qatar, Romania, Russian Federation, Sao Tome and Principe, Senegal, Serbia, Singapore, Slovakia, Slovenia, South Africa, Spain, Suriname, Sweden, Switzerland, Syrian Arab Republic, Tanzania, Timor-Leste, Trinidad and Tobago, Tunisia, Turkey, Ukraine, United Arab Emirates, United Kingdom of Great Britain and Northern Ireland, United States of America, Uruguay, Uzbekistan, Venezuela (Bolivarian Republic of), Zambia, and Zimbabwe

^c^Odds ratios were adjusted for age, country of birth, and educational level

There were lower odds of being allocated to the anal sex abstinence cluster among those aged between 25 and 34 years (aOR = 0.80; 95% CI: 0.65–0.99) and between 35 and 44 years (aOR = 0.64; 95% CI: 0.48–0.86) compared with the youngest group (18–24 years); those born in Brazil (aOR = 0.42; 95% CI: 0.29–0.62) or in other countries (aOR = 0.63; 95% CI: 0.45–0.87) compared with those born in Portugal; those who self-identified as gay (aOR = 0.59; 95% CI: 0.42–0.82); those who knew about PrEP at baseline (aOR = 0.72; 95% CI: 0.54–0.95); those who reported having a steady partner living with HIV (aOR = 0.69; 95% CI: 0.50–0.96); those who reported sex work (aOR = 0.36; 95% CI: 0.18–0.74); those who reported chemsex (aOR = 0.23; 95% CI: 0.09–0.58); and those who had an STI diagnosis (aOR = 0.83; 95% CI: 0.69–1.00).

There were lower odds of being allocated to the cluster PEP and condom use among participants aged between 35 and 44 years (aOR = 0.67; 95% CI: 0.47–0.94) and + 45 years (aOR = 0.55; 95% CI: 0.34–0.92) compared with those aged between 18 and 24 years. On the contrary, higher odds were found among participants born in Brazil (aOR = 1.90; 95% CI: 1.27–2.86) or in other countries (aOR = 1.52; 95% CI: 1.00–2.29) compared with those born in Portugal, with knowledge about PEP (aOR = 2.54; 95% CI: 1.33–4.82), who reported more than 5 occasional partners (aOR = 1.58; 95% CI: 1.35–1.85), those who had a steady partner living with HIV (aOR = 2.05; 95% CI: 1.70–2.48), those who had an occasional partner living with HIV (aOR = 3.22; 95% CI: 2.71–3.84), and those who reported sex work (aOR = 1.56; 95% CI: 1.00–2.43), chemsex (aOR = 6.23; 95% CI: 4.17–9.31), and an STI diagnosis (aOR = 2.10; 95% CI: 1.76–2.49).

Participants with higher odds of allocation to the PEP use cluster were aged between 25 and 34 years (aOR = 1.51; 95% CI: 1.17–1.96) compared with those aged between 18 and 24 years, were born in Brazil (aOR = 2.69; 95% CI: 1.79–4.05), self-identified as gay (aOR = 2.48; 95% CI: 1.18–5.21), had previous knowledge about PEP (aOR = 3.29; 95% CI: 1.11–9.77), prior knowledge about PrEP (aOR = 2.12; 95% CI: 1.71–2.62), had a steady partner living with HIV (aOR = 1.81; 95% CI: 1.41–2.30), had an occasional partner living with HIV (aOR = 1.72; 95% CI: 1.44–2.06), reported chemsex (aOR = 9.50; 95% CI: 7.25–12.44), and had an STI diagnosis (aOR = 2.09; 95% CI: 1.76–2.48). The lower odds of being allocated to this cluster were among participants aged 45 years or older (aOR = 0.54; 95% CI: 0.30–0.97), those who reported having between 1 and 5 occasional partners (aOR = 0.60; 95% CI: 0.51–0.71), and those who had engaged in sex work (aOR = 0.30; 95% CI: 0.17–0.51).

The higher odds of belonging to the cluster of PrEP and condom use were in the age groups of 25 to 34 years (aOR = 1.91; 95% CI: 1.66–2.20), 35 to 44 years (aOR = 2.78; 95% CI: 2.37–3.27), and 45 years and older (aOR = 3.11; 95% CI: 2.56–3.77), as well as among participants born in Brazil (aOR = 1.36; 95% CI: 1.16–1.59) or in other countries (aOR = 1.63; 95% CI: 1.422–1.88) when compared with those born in Portugal; those with higher education (aOR = 1.34; 95% CI: 1.19–1.52); those with previous knowledge about PEP (aOR = 1.76; 95% CI: 1.16–2.69) and PrEP (aOR = 2.90; 95% CI: 2.03–4.12); who had more than 5 occasional partners (aOR = 1.57; 95% CI: 1.29–1.92) compared with those with no occasional partners; who had an occasional partner living with HIV (aOR = 2.26; 95% CI: 1.97–2.61), reported sex work (aOR = 2.46; 95% CI: 1.82–3.31), reported chemsex (aOR = 3.48; 95% CI: 2.81–4.32), and who had an STI diagnosis (aOR = 2.79; 95% CI: 2.42–3.21).

## Discussion

Six clusters of HIV prevention were identified in this cohort of HIV-negative MSM. The condom use cluster was the most frequent, while the second most frequent cluster was low or no condom use, low PrEP Use. The other 4 clusters, related to anal sex abstinence and biomedical prevention, were much less frequent. We have also shown that PEP has been used either alone or in combination with condom use and that PrEP appeared to be associated with condom use. These combinations of HIV prevention tools find resonance in previous studies where condom use, low prevention adherence, and biomedical prevention patterns have emerged [20, 22].

It is noteworthy that despite the widespread reduction of condom use among gay, bisexual, and other MSM in recent years [30, 31], in the current study, condom use was by far the most frequently reported strategy in its cluster but also reported in other clusters with varying distribution. Even in the low or no condom use, low PrEP use cluster, condom use was reported “sometimes” in 1,910 observations and “rarely” in 1,534 observations. These results may be linked to the historical importance of condoms in the prevention of sexual transmission of HIV [32–34], being one of the most advertised and available prevention options. Furthermore, this cohort was based on testing visits to GAT CheckpointLX, which leads us to hypothesize that these participants may be more aware of risk and engaged in prevention than GAT CheckpointLX nonusers. Accordingly, other studies have shown that MSM who have an STI diagnosis tend to increase condom use [35]. In this study, having had an STI diagnosis was positively associated with being allocated to clusters that combined PrEP or PEP uptake with condom use, which could be either because participants were less protected against STI by using condoms inconsistently or because they were using condoms more often to prevent STI after a diagnosis [7, 35, 36]. Additionally, unlike other studies that have shown that MSM tend to forego condoms while taking PrEP [37], in this study, the cluster analysis did not dissociate PrEP uptake from condom use. There is, however, a lack of consensus in the literature about the influence of PrEP on condom use. Some studies have shown that when taking PrEP, MSM tend to forego condoms, while other studies have shown that taking PrEP does not impact condom use [32–34].

Over the years analyzed, the distribution of observations among clusters showed a decline in the relative importance of condom use and an increase in low or no condom use, low PrEP use, with notable stability or decreases in other clusters, except for a substantial rise in the PrEP and condom use cluster. This cluster of low or no condom use, low PrEP use was the second most relevant cluster in our analysis, and it can indicate that participants allocated to this cluster might be less engaged in prevention.

Some studies have also identified patterns of low prevention adherence, suggesting that those gay, bisexual, and other MSM do not perceive themselves as being at risk or are less concerned with HIV transmission [38]. Nevertheless, those participants might be monogamous, have partners living with HIV with suppressed viral loads, have sexual agreements with partners, or are using other tools not analyzed in this study. Research conducted in North America and Europe has shown that gay, bisexual, and other MSM engage in prevention using a vast range of strategies other than condoms and biomedical ones [36, 39–43]. Participants allocated to the low or no condom use, low PrEP use cluster reported having had a steady partner living with HIV with undetectable viral loads in 445 observations against 47 observations with reports of partners with detectable viral loads (data not shown). The same trend applies to occasional partners living with HIV with undetectable viral loads in 214 observations against 12 observations reporting partners with detectable viral loads (data not shown). They have primarily between 1 and 5 occasional partners, which could mean that participants might use condoms and PrEP only on the occasions that they do not know their partner’s serostatus and perceive a higher risk of HIV exposure. These results present, however, a 2-sided perspective because research on this topic suggests miscommunication about sexual agreements and their rules [43]. Additionally, when men break those sexual agreements, most of them avoid disclosing it to their partner(s), which could have negative implications for prevention [43]. In a similar vein, the literature reports that gay, bisexual, and other MSM also tend to decrease condom use when using other prevention strategies [22, 34, 35, 37]. Despite the importance of consistent condom use in HIV prevention, condomless intercourse is no longer equivalent to unprotected sex for HIV because the correct use of PrEP is highly effective, and people with suppressed viral loads cannot transmit HIV [38]. These results are consistent with other studies that imply that in the face of new HIV prevention options, gay, bisexual, and other MSM are not foregoing previously used tools; instead, they tend to incorporate new tools into their risk management practices and adapt them to specific contexts [35–38]. PrEP was reported in the clusters of low or no condom use, low PrEP use; anal sex abstinence; PEP and condom use; PEP use; and PrEP and condom use, despite distribution variability in each cluster, suggesting variations in PrEP uptake according to different subgroups. Nevertheless, the number of PrEP users might be underestimated because when GAT CheckpointLX users start PrEP, their follow-up related to PrEP care occurs at public hospitals or other PrEP providers. Consequently, they may discontinue GAT CheckpointLX services.

Despite educational level not being statistically significant for most clusters, participants with high education levels presented lower odds of being allocated to the low or no condom use, low PrEP use cluster and higher odds of being allocated to the PrEP and condom use cluster. This variable can be seen as a proxy of health literacy, health resources, agency, and control over prevention options that can be related to a better socioeconomic position and higher education levels that minimize obstacles to prevention and draw attention to the importance of working on education and information campaigns.

Participants with an STI diagnosis were more prone to use PEP or PrEP, which can occur because they test for STI more often during PrEP follow-up or because they forego condoms while using these strategies, being unprotected for STIs. Moreover, having an STI diagnosis is an indicator of being referred for PrEP [39]. Participants who reported having more than 5 occasional partners, who had practiced chemsex, and who had at least 1 occasional partner living with HIV also presented higher odds of using condoms, PEP, or combined PEP or PrEP with condom use, which shows an association between risk practices and risk awareness, impacting on prevention uptake. Similar results were found in other studies showing that participants meeting the criteria for PrEP referral tend to be willing to take PrEP and to be aware of their exposure to HIV [35], while other studies have shown that PrEP uptake reduces anxiety related to HIV risk [32].

The study results indicate significant potential for increasing the utilization and diversification of HIV prevention methods, particularly PrEP and PEP. Strengthening policy efforts could help improve access to these tools, encouraging broader adoption and more effective combination prevention strategies. For instance, participants in clusters with no PrEP use exhibit characteristics that suggest PrEP eligibility, such as engagement in chemsex, history of STIs, and anal sex with occasional partners living with HIV (low or no condom use, low PrEP use and PEP and condom use clusters), as well as inconsistent condom use (PEP use cluster). This indicates that expanding PrEP uptake could significantly reduce HIV transmission in these groups, as PEP is designed to be an emergency measure rather than a primary prevention tool. Moreover, despite the existence of both PEP and PrEP in Portugal, the anticipated emergence of a PEP-PrEP combination cluster did not occur, underscoring the need for an improved connection between PEP and PrEP services. The PEP2PrEP strategy remains underoptimized, suggesting that a more transparent and integrated transition between these tools is necessary to maximize their preventive potential [44].

Successful interventions in Portugal have demonstrated the value of expanding PrEP access. For example, community-based organizations have effectively increased PrEP uptake among at-risk populations, particularly within the MSM community [45]. However, there is a need to support these centers and expand their services to regions with high HIV incidence rates. Future prevention strategies aim to broaden access to PrEP and introduce new prevention tools already approved by the European Medicines Agency, such as long-acting injectable PrEP [46]. The lack of availability of these alternatives in Portugal limits access for individuals who may face challenges adhering to daily oral PrEP. Diversifying prevention methods should be accompanied by a strategic drive to better integrate PEP and PrEP within the Portuguese National Health Service. Expanding access to newer forms of PrEP, improving outreach to underserved populations (such as migrants and those with lower health literacy), and optimizing the PEP2PrEP transition are critical to reducing HIV transmission in Portugal.

As mentioned in other studies using data from the Lisbon Cohort of MSM study, it is possible to have both selection and participation bias because cohort participants tend to be born more often in Portugal and present high education levels [23]. Nevertheless, the proportion of a previous HIV test was similar between cohort participants and nonparticipants (with more diverse characteristics), suggesting a similar risk awareness that might mimic their peers on prevention utilization [23]. To minimize participation bias and the effect of differential follow-up losses through time, a sensitivity analysis (data not shown) considering only information from the first visit showed that the clusters would have a similar configuration, suggesting that the clusters described represent the GAT CheckpointLX target population. Participants also reported more frequently having been born in Portugal, having high education levels, and self-identifying as gay. Nevertheless, and despite these limitations, our study uses data from a well-established cohort study, allowing for longitudinal analysis focusing on prevention in an HIV key population.

This study might inform health programs and policies regarding HIV prevention because understanding behaviors and characteristics associated with prevention can help to improve and design tailored health interventions. Still, we consider that to understand this topic more comprehensively, there is a need for more studies about behavior and risk perceptions, longitudinal studies, and in-depth studies about sexual agreements and relationships. Moreover, there needs to be an effort to reach those less engaged in participating in these studies to have more diversified samples. In conclusion, we showed that participants in this cohort of MSM rely mostly on condoms for HIV prevention. We also showed that the potential for prevention combination and using PrEP may still be optimized through increasing awareness and access to all the available prevention options.

## Data Availability

The data supporting this study’s findings are available from the corresponding author upon reasonable request.
